# Attenuation of primate aging via systemic infusion of senescence-resistant mesenchymal progenitor cells

**DOI:** 10.1186/s13619-025-00248-8

**Published:** 2025-06-27

**Authors:** Aisha Siddique, Ismail M. Shakir, Mo Li

**Affiliations:** 1 Bioscience Program, Biological and Environmental Science and Engineering Division (BESE), King Abdullah University of Science and Technology (KAUST), Thuwal, 23955 Saudi Arabia; 2 KAUST Center of Excellence for Smart Health (KCSH), Thuwal, 23955 Saudi Arabia

## Abstract

Aging is characterized by progressive functional decline driven by stem cell exhaustion, chronic inflammation, and cellular senescence. Mesenchymal progenitor cells (MPCs), which play a central role in tissue repair, are particularly vulnerable to age-associated dysfunction. Lei et al. (Cell 188:1-22, 2025) address this limitation by engineering human embryonic stem cell-derived MPCs with enhanced FOXO3 activity (termed SRCs). Intravenous administration of FOXO3-SRCs to aged cynomolgus macaques significantly slowed aging across multiple organs compared to wild-type MPCs. SRC treatment improved cognitive performance, preserved brain structure, protected bone integrity, and rejuvenated immune function. Transcriptomic and DNA methylation aging clocks revealed substantial reductions in biological age, with the most pronounced rejuvenation observed in the reproductive system, skin, lung, muscle, and hippocampus. These effects were partly attributed to SRC-derived exosomes enriched in gero-protective proteins and metabolites. Importantly, SRCs exhibited robust safety, showing no tumorigenicity or immunogenicity. This work positions FOXO3-enhanced MPCs and their exosomes as promising candidates for systemic anti-aging interventions, shifting the therapeutic paradigm from treating individual diseases to targeting the aging process itself.

## Main text

Aging, marked by progressive functional decline across organ systems, is driven by stem cell exhaustion, chronic inflammation, and cellular senescence. Human mesenchymal progenitor cells (MPCs), including mesenchymal stem cells (MSCs), play critical roles in tissue repair and regeneration but exhibit functional decline with aging due to cellular senescence and microenvironmental changes (Chen et al. 2022). Recent advances in genetic engineering have opened avenues to enhance cellular resilience against age-related stressors. An earlier study by Yang et al. (2017) showed that a single-nucleotide recoding of the *NRF2* gene in human embryonic stem cells (hESCs) generated MSCs with enhanced regenerative capacity and resistance to aging and tumor formation (Yang et al. 2017). Recent work by the same group demonstrated that FOXO3-engineered hESC-derived vascular cells exhibit superior resistance to oxidative injury and delayed aging in mouse models, establishing the activation of the transcription factor FOXO3 as a key strategy for cellular rejuvenation (Yan et al. 2019). Following this, Zhang et al. (2020) mapped primate arterial aging at single-cell resolution, identifying FOXO3 downregulation as a hallmark of vascular aging and implicating inflammatory pathways as therapeutic targets (Zhang et al. 2020). Population studies further corroborated FOXO3's role in human longevity, with polymorphisms linked to extended lifespans and reduced age-related disease incidence (Morris et al. 2015).

Drawing from these prior findings (Yan et al. 2019; Zhang et al. 2020), Lei et al. (2025) in their current work demonstrated that intravenous (IV) administration of FOXO3-enhanced seno-resistant human MPCs (SRCs) proved more effective than wild-type MPCs (WTCs) at slowing the progression of aging across multiple organs in aged cynomolgus macaques. They showed that the systemic infusion of FOXO3-SRCs induced measurable improvements in brain function, bone density, and reproductive health with mechanistic analyses revealing these therapeutic effects were partly mediated through SRC-derived exosomes (Lei et al. 2025).

**Fig. 1:**
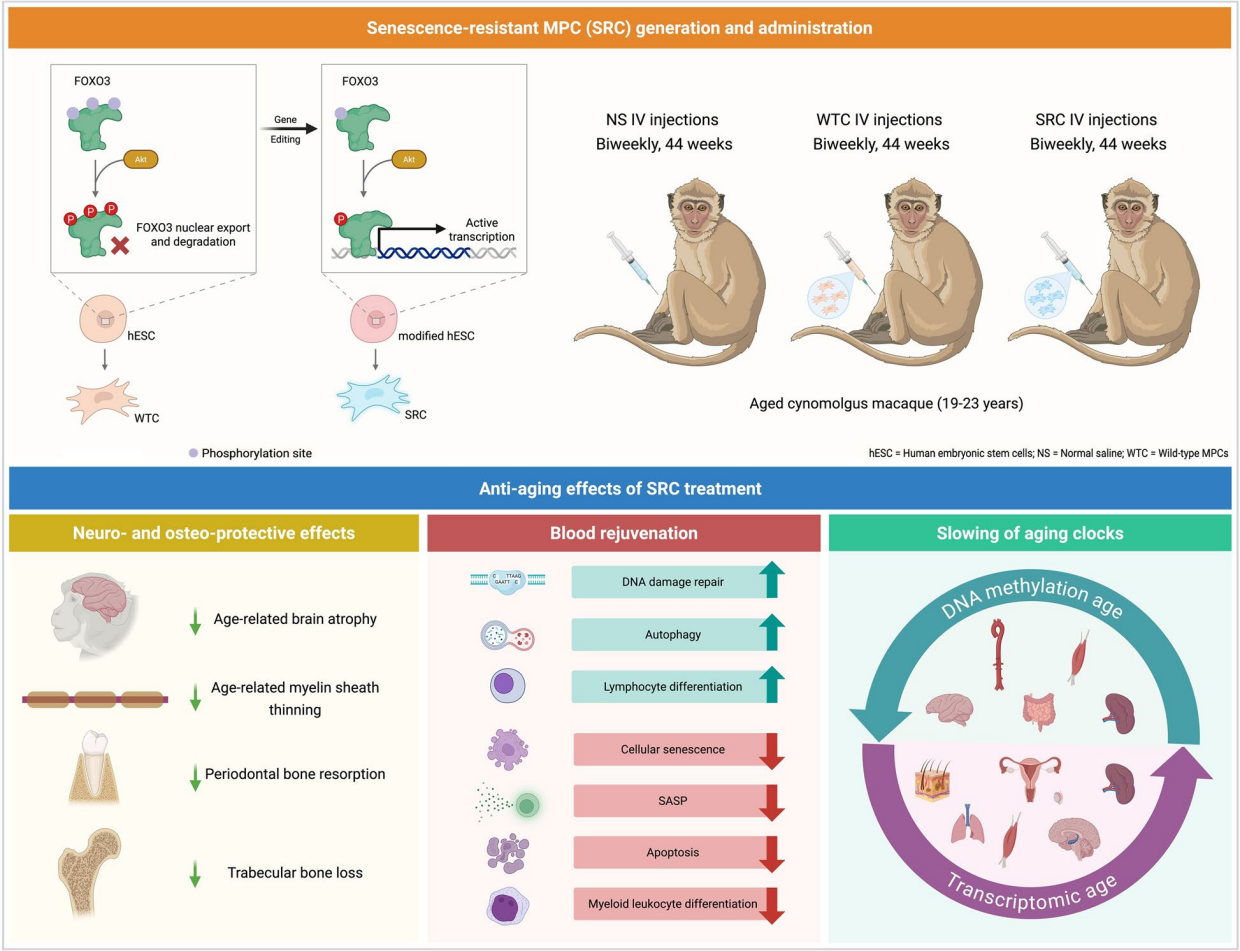
Study by Lei et al. (2025) demonstrates that administration of FOXO3-enhanced SRC to aged macaques reverses the aging clock and rejuvenates multi-organ health. hESCs are gene edited to enhance FOXO3 expression and then reprogrammed to MPCs, termed SRCs. These SRCs when administered to aged cynomolgus macaques, for a period of 44 weeks, result in reducing effects of aging in various organs compared to untreated (control) macaques. Both transcriptomic and epigenetic aging clocks of the SRC-treated macaques are slowed down, mainly in the organs depicted in the figure (right bottom panel) (Lei et al. 2025)

In their study, Lei et al. (2025) addressed a central limitation of traditional MPC therapies–their vulnerability to the inflammatory and oxidative stress-rich environment of aged tissues, which often leads to rapid cell deterioration and limited clinical benefit. Building upon previous findings that FOXO3 activity declines in aged primate tissues, the authors used precise gene editing to create hESC-derived MPCs with biallelic knock-in mutations (FOXO3 p.[Ser253Ala;Ser315Ala]) that enhance FOXO3 activity (Fig. 1). These SRCs exhibited robust nuclear accumulation of FOXO3, increased transcriptional activity, and a suite of youthful cellular traits, including reduced senescence-associated beta-galactosidase (SA-β-Gal) activity, extended telomeres, improved heterochromatin stability, and diminished expression of senescence-associated secretory phenotype (SASP) factors. Importantly, SRCs demonstrated enhanced resistance to oxidative and genotoxic stressors, maintained genomic stability, and showed no evidence of tumorigenicity in transplantation assays. Consequently, these properties positioned SRCs as a promising, clinically relevant cell product for anti-aging interventions in primates (Lei et al. 2025).

In a 44-week preclinical trial, aged cynomolgus macaques were administered IV infusions of SRCs, WTCs, or saline. Interestingly, the SRC-treated group exhibited minimal adverse effects, with no evidence of immunogenicity or tumor formation, confirming the safety of long-term, repeated administration. The therapeutic efficacy of SRCs was evaluated through a battery of physiological, imaging, and molecular assays (Lei et al. 2025).

Systemic SRC treatment led to improved performance in memory retention tasks compared to controls, indicating a potential cognitive benefit. MRI-based analyses revealed preservation of cortical thickness and increased volume in the frontal and parietal lobes, regions susceptible to age-related atrophy. Diffusion MRI and functional connectivity analyses showed enhanced structural and functional connectivity in aging-affected brain regions, including the hippocampus. Furthermore, electron microscopy demonstrated that SRCs ameliorated age-associated myelin sheath thinning, while histological analyses confirmed increased expression of myelin basic protein, suggesting improved neural integrity. Additionally, micro-CT imaging showed that SRC-treated macaques experienced less age-related periodontal bone loss and reduced degradation of trabecular bone structure, indicating a protective effect against osteoporosis and skeletal degeneration (Fig. 1) (Lei et al. 2025).

Interestingly, single-cell transcriptomic analyses of peripheral blood mononuclear cells (PBMCs) revealed that SRCs more effectively reversed age-related gene expression changes compared to WTCs, particularly those associated with immune response, cellular senescence, and apoptosis. SRC treatment upregulated genes involved in DNA repair, autophagy, and lymphocyte differentiation, supporting the rejuvenation of immune cell function (Fig. 1). Markers of cellular senescence, DNA damage, and inflammation were consistently reduced in PBMCs and plasma, with decreased levels of SASP factors, such as IL-6 and TNF-α. Furthermore, SRCs also reduced CHIT1 in cerebrospinal fluid, a biomarker of neuroinflammation (Lei et al. 2025).

Using machine-learning-based transcriptomic and DNA methylation aging clocks, Lei et al. quantified biological age reductions across 61 tissues from 10 organ systems. SRC treatment reduced transcriptomic age by an average of 3.34 years in 54% of tissues, with parallel reductions in DNA methylation age (Fig. 1). The most pronounced rejuvenation was observed in the reproductive system, followed by the skin, lung, skeletal muscle, and hippocampus, which were confirmed by histological analyses that showed reduced senescence markers and restoration of youthful nuclear architecture (marked by Lamin B1 and H3K9me3) (Lei et al. 2025).

Investigating the mechanisms through which SRCs systemically attenuate aging presented a critical yet complex challenge. The authors tackled this challenge by hypothesizing that SRC-derived exosomes (SRC-Exo) serve as mediators of these anti-aging effects. Proteomic and metabolomic analyses revealed that SRC-Exo are enriched in gero-protective proteins (antioxidants, anti-inflammatory factors, and modulators of innate immune responses) and metabolites such as spermine–known to reverse the effects of aging in aged-mice brains (Xu et al. 2020).

To further substantiate the functional significance of exosomes, Lei et al. demonstrated that in aged mice, IV SRC-Exo administration delayed transcriptomic aging across multiple organs and reduced senescent cell burden. In vitro, SRC-Exo reversed senescence markers in human neurons, ovarian stromal and endothelial cells, aortic endothelial cells, and hepatocytes. Importantly, at equivalent concentrations, SRC-Exo consistently outperformed exosomes from WTCs, supporting their superior gero-protective potential. These results give credence to the idea that paracrine signaling via exosomes is a mechanism by which SRCs exert systemic rejuvenation (Lei et al. 2025).

The study by Lei et al. made significant strides in overcoming major obstacles to the clinical application of MPCs in therapy. First, the authors engineered FOXO3-SRCs that demonstrated a strong safety profile in non-human primates, exhibiting neither tumorigenicity nor immune rejection even after extended administration. Second, they identified quantitative biomarkers, such as multi-tissue aging clocks, which allow for precise and objective monitoring of therapeutic efficacy over time. Despite these advances, important questions remain: (a) whether the observed systemic rejuvenation arises directly from dose-dependent effects of SRC-Exo or indirectly via the amplification of rejuvenated endogenous tissue responses; (b) whether the in vivo biodistribution of SRCs mirrors their rejuvenation effects; and (c) whether certain organ systems or tissues—such as the hematopoietic system—hold therapeutic priority and can function as central hubs for systemic rejuvenation. Addressing these gaps will be essential for informing future research aimed at establishing the optimal dosage, timing, and mode of administration for allogenic SRC/SRC-Exo in human studies. While challenges remain in optimizing delivery regimens, this work shifts the paradigm from treating isolated age-related diseases to systemically targeting aging itself.

## Conclusions

In summary, Lei et al. (2025) demonstrate that systemic administration of FOXO3-enhanced seno-resistant human MPCs safely and effectively slows multi-organ aging in primates, largely through exosome-mediated mechanisms. Their findings establish a promising foundation for future clinical application of engineered cell therapies for aging.

## Data Availability

Not applicable.
